# Redox-Dependent Chromatin Remodeling: A New Function of Nitric Oxide as Architect of Chromatin Structure in Plants

**DOI:** 10.3389/fpls.2019.00625

**Published:** 2019-05-28

**Authors:** Alexandra Ageeva-Kieferle, Eva Esther Rudolf, Christian Lindermayr

**Affiliations:** Institute of Biochemical Plant Pathology, Helmholtz Zentrum München – German Research Center for Environmental Health, Munich, Germany

**Keywords:** nitric oxide, redox-modification, S-nitrosation, chromatin modulation, acetylation, methylation

## Abstract

Nitric oxide (NO) is a key signaling molecule in all kingdoms. In plants, NO is involved in the regulation of various processes of growth and development as well as biotic and abiotic stress response. It mainly acts by modifying protein cysteine or tyrosine residues or by interacting with protein bound transition metals. Thereby, the modification of cysteine residues known as protein S-nitrosation is the predominant mechanism for transduction of NO bioactivity. Histone acetylation on N-terminal lysine residues is a very important epigenetic regulatory mechanism. The transfer of acetyl groups from acetyl-coenzyme A on histone lysine residues is catalyzed by histone acetyltransferases. This modification neutralizes the positive charge of the lysine residue and results in a loose structure of the chromatin accessible for the transcriptional machinery. Histone deacetylases, in contrast, remove the acetyl group of histone tails resulting in condensed chromatin with reduced gene expression activity. In plants, the histone acetylation level is regulated by S-nitrosation. NO inhibits HDA complexes resulting in enhanced histone acetylation and promoting a supportive chromatin state for expression of genes. Moreover, methylation of histone tails and DNA are important epigenetic modifications, too. Interestingly, methyltransferases and demethylases are described as targets for redox molecules in several biological systems suggesting that these types of chromatin modifications are also regulated by NO. In this review article, we will focus on redox-regulation of histone acetylation/methylation and DNA methylation in plants, discuss the consequences on the structural level and give an overview where NO can act to modulate chromatin structure.

## Sources and Intracellular Localization of Nitric Oxide

In plants, NO is formed either by reductive or oxidative pathways. In mammals, three cell-specific NO synthases (NOS) oxidize arginine to citrulline, thereby releasing NO. Although NOS-like activities have been measured in chloroplasts and peroxisomes of higher plants (He et al., 2004; Corpas and Barroso, 2018), NO synthase has only been identified in the algae (Foresi et al., 2010). Other possible substrates for oxidative NO production involve polyamines and hydroxylamine (Groß et al., 2013; Farnese et al., 2016). Reduction of nitrite to NO constitutes the reductive route of NO production (Rockel et al., 2002; Yamamoto-Katou et al., 2006; Srivastava et al., 2009). Usually, nitrate reductase catalyzes the reduction of nitrate to nitrite. However, under low oxygen conditions, light and high nitrite levels, nitrite can be reduced to NO (Rockel et al., 2002; Planchet et al., 2005). Finally, enzyme-independent reduction of nitrite has been described in apoplast under acidic conditions (Bethke et al., 2004). Intracellular sources of NO are located in various compartments, including cytosol, peroxisomes, mitochondria, and chloroplasts (summarized in Groß et al., 2013; Farnese et al., 2016). Nuclear NO production is not described in plants. However, thiol reducing systems like thioredoxins and glutaredoxins as well as reducing molecules such as glutathione were found in the nucleus, suggesting that thiol modifications occur in this compartment (Delorme-Hinoux et al., 2016; Martins et al., 2018). Because of its lipophilic character, NO can easily cross the nuclear membrane or enter *via* nuclear pores (Toledo and Augusto, 2012; Lancaster, 2015). Moreover, NO can be transferred into the nucleus *via* S-nitrosylated proteins or S-nitrosylated low molecular weight thiols, such as S-nitrosoglutathione (GSNO) or S-nitrosocysteine. S-Nitrosylated nuclear proteins have been identified using the biotin switch technique, which labels S-nitrosylated proteins with a biotin linker allowing detection, purification, and identification of these proteins (Chaki et al., 2015). In mammals, nuclear translocation of S-nitrosylated proteins is described for gylceralaldehyd-3-phosphat-dehydrogenase and chloride intracellular channel protein CLIC4 (Hara et al., 2005; Malik et al., 2010). Nuclear localization of gylceralaldehyd-3-phosphat-dehydrogenase has been characterized in *Arabidopsis* (Holtgrefe et al., 2008; Vescovi et al., 2013; Aroca et al., 2017).

## Physiological Function and Biochemistry of Nitric Oxide

The chemical properties of nitric oxide (NO) make it highly multifunctional. Whereas some studies report toxic and harmful action of NO species, such as cell death (Pedroso et al., 2000), damage of proteins, membranes, and nucleic acids, or photosynthetic inhibition (Yamasaki, 2000), others demonstrate protective and/or signaling function of NO species. In fact, the dual function of NO is often dependent on its concentration and environment. Based on its functions, NO has been proposed as a stress-responding agent. It can counteract toxic processes induced by ROS (Beligni and Lamattina, 1998; Sun et al., 2007). It was shown that NO is involved in abiotic stress responses such as salinity, drought, UV-B radiation, temperature, and heavy metal toxicity (Mata and Lamattina, 2001; Tian et al., 2007). The role of NO in biotic stress is essential. It plays a key role in disease resistance against *Pseudomonas syringae* in *Arabidopsis* leaves, and is required for SAR induction in tobacco (Delledonne et al., 1998; Hong et al., 2008). Moreover, NO participates in plant development and physiological processes such as germination, gravitropism, root development, and flowering (Correa-Aragunde et al., 2004; He et al., 2004; Zhang et al., 2005). Although there is no doubt that NO is crucial for plant development and survival, the mechanism by which NO activates signaling function and the genes underlying this process remain to be elucidated.

NO chemical properties contribute to its role in signal transduction in a living cell (Toledo and Augusto, 2012; Lancaster, 2015). It can rapidly undergo multiple chemical reactions with enzymes, transcription factors, second messengers, or chromatin modifiers (Yu et al., 2014; Kovacs et al., 2016a). NO and its related species are able to modulate protein activities and biological function through covalent post-translational modifications (PTM) by binding to the metal centers of proteins and by affecting their cysteine and tyrosine residues. Tyrosine nitration is a post-translational modification that arises through the binding of a NO_2_ into ortho carbons of aromatic ring of tyrosine residues that leads to the formation of 3-nitrotyrosine (Mata-Pérez et al., 2016; Kolbert et al., 2017). In a direct reaction termed metal nitrosylation, NO binds to transition metals, resulting in formation of metal nitrosyl complexes. In this way, activity and function of proteins can be regulated. Well studied targets for NO interaction are iron-sulfur clusters, as well as heme groups and zinc ions of proteins (Astier et al., 2010).

Examples of NO binding to iron present in heme proteins have also been observed in plants. It was suggested that two major H_2_O_2_-scavenging enzymes in tobacco, ascorbate peroxidase, and catalase are reversible inhibited by NO donors through the formation of an iron-nitrosyl complex (Clark et al., 2000). Plant hemoglobins were also identified as a target for NO. It was shown that *Arabidopsis* nonsymbiotic hemoglobin AHb1 binds NO and oxidizes it to nitrate, suggesting a role of hemoglobins in detoxification of NO (Perazzolli et al., 2004; Kuruthukulangarakoola et al., 2017). S-Nitrosation is the most studied redox-based post-translational modification. This modification results in the formation of S-nitrosothiols (SNO). S-Nitrosation enables a living organism to directly respond to environmental stimulus through the regulation of protein activity, protein-protein interaction, or protein localization (Hara et al., 2006; Yun et al., 2011). The release of the NO moiety from proteins and therefore the control of SNO homeostasis in a cell is maintained by two enzymes: GSNOR reductase (GSNOR), which metabolizes GSNO to a mixture of intermediates, and thioredoxins, which mediate denitrosylation (López-Sánchez et al., 2008; Cañas et al., 2012; Kneeshaw et al., 2014). Furthermore, reduced glutathione (GSH) alone is able to denitrosylate S-nitrosylated proteins. For instance, physiological levels of GSH rapidly removed the NO moiety of S-nitrosylated GAPDH resulting in the reduced and active form of GAPDH (Zaffagnini et al., 2013). For this GSH-dependent protein denitrosylation, the GSH/GSNO ratio is of relevance, but not the GSH/GSSG ratio. Although a high number of candidates for S-nitrosation were identified, only a few of them were experimentally confirmed and their functions in response to NO demonstrated (Astier et al., 2012). Most of the studies are based on biotin switch technique, where S-nitrosated cysteines are labeled with a biotinylating agent, allowing easy detection by immunoblotting using anti-biotin antibodies or purification using streptavidin matrix.

Interestingly, S-nitrosation of transcription factors can affect their function. For instance, S-nitrosation of Cys53 of the Arabidopsis R2R3-MYB2 transcription factor inhibited its DNA-binding activity (Serpa et al., 2007). Similarly, S-nitrosation of Cys49 and Cys53 of MYB30 results are structural changes negatively affecting DNA affinity of this transcription factor (Tavares et al., 2014). In contrast, S-nitrosation of TGA1, a transcription factor involved in the activation of pathogenesis-related (PR) genes, promotes the binding to the as1-element of the PR1 promoter (Lindermayr et al., 2010). Besides the regulatory function of NO on transcription factors, NO can control gene transcription also by affecting chromatin structure and/or DNA accessibility.

## Regulation of Histone Acetylation/Methylation and DNA Methylation by Nitric Oxide

On the genome level (nucleotide sequence), cells of multicellular organisms are identical. However, each cell differs from others through the differences in gene expression pattern that might occur in a temporally and spatially-dependent manner. Genes might be silenced/switched off and switched on again only when they are required. Such an activation/inactivation can be regulated through a direct control of regulatory elements on gene promoters. Moreover, over the last few decades, it was found that affecting the accessibility of the DNA by modification of the chromatin structure, is also a key regulator of transcription.

Genetic material of all eukaryotic organisms has to be packed into the nucleus to prevent it from becoming damaged. Since the length of eukaryotic DNA is far greater than the diameter of a nucleus, it has to be organized in a very tightly packaged structure, known as chromatin. The core subunit of chromatin is an octamer, which is composed of two copies of the histone proteins, H2A, H2B, H3, and H4, which are positively charged and enable an electrostatic interaction with negatively charged DNA. 145–147 bp of DNA are wrapped around a histone complex forming repeating nucleosomal units, which are connected with each other by short DNA fragments called “linker DNA.” Linker histone H1 is located between the nucleosomes and stabilizes chromatin structure, resulting in highly condensed 30 nm fibers (Luger et al., 2012).

Chromatin structure in eukaryotic organisms is very dynamic, and can be changed during growth and development and in response to environmental stimuli. Chromatin marks are able to induce chromatin remodeling and therefore to control important molecular processes such as gene transcription, replication, repair, and recombination (Bannister and Kouzarides, 2011). DNA methylation and histone modifications are the key mediators of epigenetic modifications. DNA methylation is usually associated with long-term silencing of genes, whereas histone modifications contribute to both activation and repression of gene transcription and can be removed after several cell cycles (Jaenisch and Bird, 2003; Minard et al., 2009).

Histone modifications play an important role in the regulation of chromatin structure and in subsequent gene transcription. N-terminal histone tails, which are exposed outside the nucleosome may interact with neighboring nucleosomes and therefore manipulate the chromatin structure (Bannister and Kouzarides, 2011). Histone tails can undergo different posttranslational modifications such as acetylation, methylation, phosphorylation, and ubiquitination. These can act alone or in combination, resulting in different molecular changes that effect DNA accessibility.

### Nitric Oxide Inhibits Histone Deacetylases

Histone acetylation plays a key role in regulation of gene transcription (Servet et al., 2010; Shen et al., 2015). This modification is very dynamic and is catalyzed by two families of enzymes: histone acetyltransferases (HATs) and histone deacetylases (HDAs). The transfer of acetyl groups from acetyl-coenzyme A on histone lysine residues is catalyzed by histone acetyltransferases. This modification neutralizes the positive charge of the lysine residue and reduces the interaction between histones and the negatively charged DNA (Schiedel and Conway, 2018). This results in a loose chromatin structure accessible for DNA binding proteins. Histone deacetylases, in contrast, remove the acetyl group of histone tails resulting in condensed chromatin with reduced gene expression activity (Hollender and Liu, 2008; Luo et al., 2012). Therefore, histone acetylation is usually associated with gene transcription. For instance, differential acetylation at H3K9 and H3K27 and phosphorylation at H3S28 between end-of-night and end-of-day correlates with changes in diurnal transcript levels of core clock genes in *Arabidopsis* (Baerenfaller et al., 2016). In poplar, expression of carbonic anhydrase, pyruvate orthophosphate dikinase, phosphoenolpyruvate carboxykinase, and phosphoenolpyruvate carboxylase correlates with acetylation of H3K9 and H4K5 at their promoter regions (Li et al., 2017). Moreover, deacetylation of the flowering gene *AGL19* represses its transcription (Kim et al., 2013). However, there are also examples where enhanced acetylation of nearby regulatory elements and coding sequences does not generally result in higher transcription of the corresponding gene (Mengel et al., 2017). For example, comparison of ChIP-seq and transcript data of genes displaying GSNO-regulated H3K9/14 ac demonstrated that the mRNA levels of more than 60% of these genes remained unchanged (Mengel et al., 2017), concluding that histone acetylation is indeed making DNA accessible, but does not *per se* leads directly to gene transcription.

There is increasing evidence that the catalytic activity of at least some HDAs is regulated by redox modifications, which are involved in the regulation of unwinding and wrapping of chromatin. Until now, most studies of redox regulation by HDAs have been done in human and animal cells. It was reported that HDA2 in neurons gets S-nitrosated upon NO signaling triggered by brain-derived neurotrophic factor (Nott et al., 2008). S-Nitrosation of HDA2 results in chromatin acetylation and activation of gene expression that are involved in neuronal development. Notably, S-nitrosation does not affect the enzymatic activity of HDA2, but stimulates its release from chromatin. Influence of NO on HDA2 was confirmed, when redox-sensitive cysteines were mutated to alanine preventing dissociation of HDA2 from chromatin (Nott et al., 2008). S-Nitrosation of HDA2 was also demonstrated in muscles of dystrophin-deficient MDX mice (Colussi et al., 2008). The catalytic activity of this enzyme was impaired by NO *in vivo* and *in vitro.* Additionally, protein activity in the presence of NO was also measured in purified *Escherichia coli* produced HDA1, HDA2, and HDA3. Recombinant HDA2 was highly sensitive to NO donors, and a slight reduction of protein activity was measured in HDA1 that was not caused by S-nitrosation (Colussi et al., 2008). Human HDA6 and HDA8 were also identified as potential targets for NO (Feng et al., 2001; Okuda et al., 2015). Endogenous HDA6 was identified as target for S-nitrosation using the biotin switch assay (Okuda et al., 2015). S-Nitrosation of HDA6 inhibited its catalytic activity and increased the level of acetylated alpha-tubulin suggesting that HDA6 plays a crucial regulatory function in acetylation of proteins others than histones (Okuda et al., 2015). HDA8 is S-nitrosated by GSNO *in vitro* (Feng et al., 2001). Moreover, the protein activity was significantly reduced by GSNO and another NO donor, S-nitrosocysteine, in time- and concentration-dependent manner. Interestingly, application of the NO donor sodium nitroprusside (SNP) to HDA8 had no effect on the catalytic activity of this protein, indicating that a special structural interaction is required for transferring NO (Feng et al., 2001). NO-dependent inhibition of gene expression was measured in human umbilical vein endothelial cells (Illi et al., 2008). It was demonstrated that upon NO production, protein phosphatase becomes activated and associates with a histone deacetylase complex pCamkIV/HDAs, promoting its dephosphorylation. This process leads to the shuttling of HDA4 and HDA5 (members of pCamkIV/HDAs complex) to the nucleus and deacetylation of histones. As a consequence, c-fos gene expression is inhibited. c-fos encodes for a protein with a basic leucine zipper region for dimerization and DNA-binding and a C-terminal transactivation domain (Illi et al., 2008). It is involved in important cellular events, including cell proliferation, differentiation and survival. Under non-stressed conditions, when the NO level in the cell is low, HDA4 and HDA5 remain in the cytosol, allowing hyperacetylation of chromatin (Illi et al., 2008). If similar mechanisms exist in plants as well still has to be investigated.

In *Arabidopsis* there are 18 members of HDAs, which are divided into 3 families: RPD3-like, HD-tuins, and sirtuins (Hollender and Liu, 2008; König et al., 2014; Shen et al., 2015; Bourque et al., 2016). The first family is the largest one and is composed of 12 putative members (HDA2, HDA5-10, HDA14-15, HDA17-19), which, based on their structure, can be further divided into 3 subclasses. This family of HDAs is homologous to yeast reduced potassium deficiency 3 (RPD3) proteins that are present across all eukaryotes (Hollender and Liu, 2008). All members of this family contain a specific deacetylase domain that is required for their catalytic activity. It should be highlighted that this class of HDAs is able to deacetylate more targets than just histones. Lysine acetylome profiling uncovered 91 acetylated proteins in *Arabidopsis* leaves after the treatment with deacetylase inhibitors apicidin and trichostatin A. Of these, only 14 were histone-like proteins (Hartl et al., 2017). The second family is plant-specific and contains the HD-tuins (HD2). These type of proteins was originally found in maize. The amino acid sequence of HD-tuins is related to cis-trans prolyl isomerases, which are present in other eukaryotes (Aravind and Koonin, 1998; Bourque et al., 2016). HD2s are structurally distinct from RPD3-like members, but display a sequence similarity with FK506-binding proteins. In total, four members of HD-tuins have been identified in *Arabidopsis*: HDT1 (HD2A), HDT2 (HD2B), HDT3 (HD2C), and HDT4 (HD2D). These consist of an N-terminal domain that has a conserved pentapeptide MEFWG region, which is part of a gene repression activity (Bourque et al., 2016). This region is followed by a high-charged acidic motif that is rich in glutamic and/or aspartic acid and a variable C-terminal region (Dangl et al., 2001). Moreover, HDT1 and HDT3 possess a zinc-finger motif that probably is involved in protein-protein interaction and DNA-binding (Bourque et al., 2016). The third family of plant HDAs is represented by sirtuins (SIR2-like proteins), which are homologs to yeast silent information regulator 2 (SIR2) (König et al., 2014; Bheda et al., 2016). These HDAs are unique because they require a NAD cofactor for their function and unlike RPD3 proteins, they are not inhibited by trichostatin A or sodium butyrate. Moreover, sirtuins use a wide variety of substrates beyond histones (König et al., 2014; Bheda et al., 2016).

**Figure 1:**
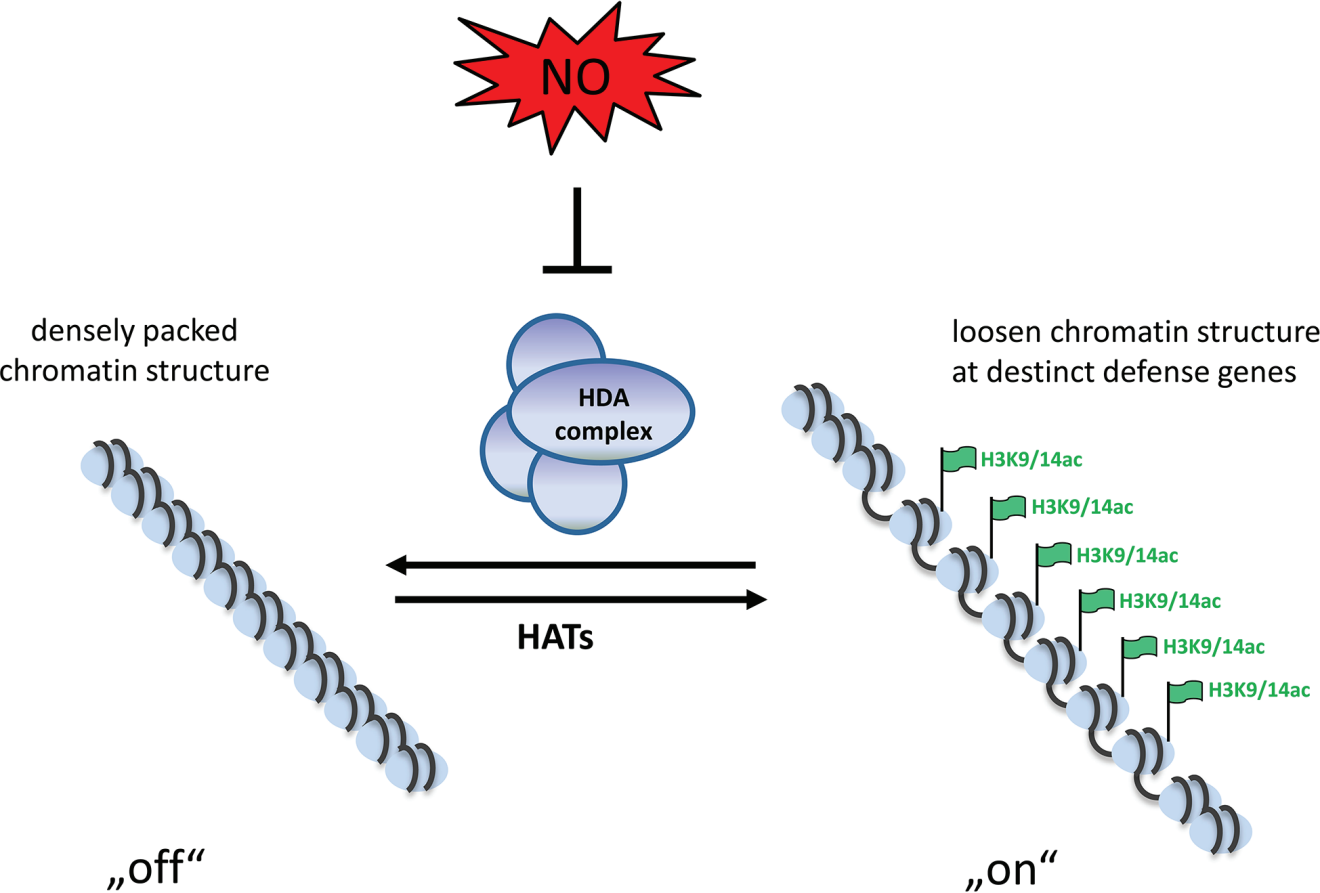
Schematic model of NO-induced chromatin modulation. Due to the activity of HDAs the chromatin is densely packed and genes are not transcribed. Upon formation of NO, the HDA-complexes become inhibited by S-nitrosation, leading to acetylation of the chromatin. This loosen chromatin structure allows transcription in tight interplay with activating transcription factors.

Similar as in humans/animals redox molecules modulate histone acetylation in plants, too. Two members of the plant RPD-3 like family (HDA9 and HDA19) are sensitive to oxidation; however, the physiological function of this modification is still not understood (Liu et al., 2015). Treatment of *Arabidopsis* seedlings with the physiological NO-donor GSNO increased the abundance of several histone 3 and histone 4 acetylation marks. Presence of the NO scavenger 2-4-carboxyphenyl-4,4,5,5-tetramethylimidazoline-1-oxyl-3-oxide (cPTIO) strongly diminished the abundance of these histone mark (Mengel et al., 2017). Since, GSNO and S-nitroso-N-acetyl-DL-penicillamine (SNAP) reversibly reduced total HDA activity both *in vitro* and *in vivo*, the increased acetylation was likely caused by NO-dependent inhibition of HDA activity. Moreover, the major plant defense hormone salicylic acid, inhibited HDA activity and increased histone acetylation by inducing endogenous NO production. Additionally, genome-wide NO-dependent H3K9/14 ac profiling in *Arabidopsis* seedlings identified NO-regulated histone acetylation of genes involved in plant defense response and abiotic stress response. This includes, for example, genes encoding for TIR class nucleotide-binding site-leucine-rich repeat (TIR-NBS-LRR) class disease resistance proteins and the transcription factors WRKY27, WRKY53, TGA2 and TGA5 (Mengel et al., 2017). Plant proteins belonging to the nucleotide-binding site-leucine-rich repeat (NBS-LRR) family are used for pathogen detection. These proteins detect pathogen-associated proteins, such as the effector molecules responsible for virulence. The TIR class of plant NBS-LRR proteins contains an additional amino-terminal domain homolog to the Toll and interleukin 1 receptors (DeYoung and Innes, 2006). WRKY transcription factors are key players in modulating the transcriptome during plant defense response. WRKY27 controls the expression of genes involved in nitrogen metabolism and NO production and negatively influences symptom development of *Ralstonia solanacearum* in *Arabidopsis* (Mukhtar et al., 2008). WRKY53 acts in a transcription factor signaling network mediating together with the EPITHIOSPECIFYING SENESCENCE REGULATOR a negative crosstalk between pathogen resistance and senescence, most likely controlled by the equilibrium between jasmonic acid and salicylic acid (Miao and Zentgraf (2007). TGA2 or TGA5 simultaneously bind to the TGACG motif of the *Pathogenesis-related1* promoter activating expression of this defense gene (Zhang et al., 2003; Hussain et al., 2018). In sum, NO regulates histone acetylation by modifying and inhibiting HDA complexes. This results in hyperacetylation of specific genes enabling their transcription. This might be an important mechanism operating in the plant stress response and facilitating expression of stress-related genes (Figure 1; Mengel et al., 2017).

**Figure 2:**
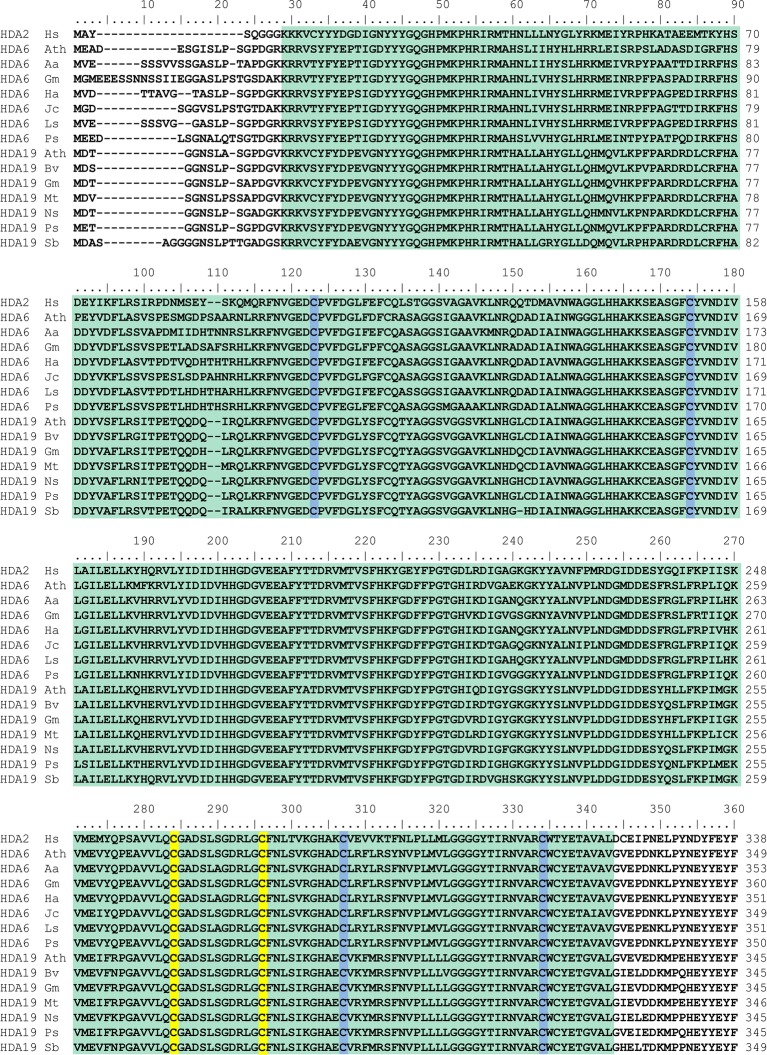
Alignment of the amino acid sequences of the HDA domain of human HDA2 and different plant histone deacetylase 6 and 19 proteins. HDA amino acid sequences were aligned using Clustal W. The HDA domain is depicted in green. Cysteine residues of human HDA2 which are targets for S-nitrosation and the corresponding cysteine residues of plant HDAs are highlighted in yellow. Other conserved cysteine residues are marked in blue. Hs, *Homo sapiens* NP_001518.3; Ath, *Arabidospsis thaliana* AED97705.1 (HDA6) and O22446.2 (HDA19); Aa, *Artemisia annua* PWA92260.1; Gm, *Glycine max* XP_003525556.1 (HDA6) and XP_003543935.1 (HDA19); Ha, *Helianthus annuus* XP_021978414.1; Jc, *Jatropha curcas* XP_012079994.1; Ls, *Lactuca sativa* XP_023740973.1; Ps, *Papaver somniferum* XP_026387130.1 (HDA6) and XP_026455725.1 (HDA19); Bv, *Beta vulgaris* XP_010690952.1; Mt., *Medicago truncatula* XP_013462369.1; Ns, *Nicotiana sylvestris* XP_009770456.1; Sb, *Sorghum bicolor* XP_002438614.1.

**Figure 3:**
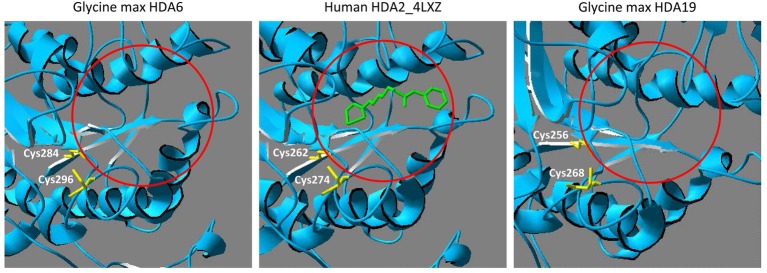
Comparison of HDA6, HDA19 and human HDA2 substrate binding site. Structural comparison between human HDA2 and *Glycine max* HDA6 and HDA19. The HDA domains of *G. max* HDA6 (amino acids 29–397, Uniprot entry I1MTD8) and of *G. max* HDA19 (amino acids 3–371, Uniprot entry A0A0R0H2W2) were modeled using the SwissProt Modeling server with human HDA2 as template (PDB entry 4LXZ). The histone deacetylase inhibitor octanedioic acid hydroxyamide phenylamide is highlighted in green and shows the location of the active site (mark with a red circle). Cysteine residues, which are located next to the active site are marked in yellow. These cysteine residues are targets for S-nitrosation in human HDA2.

All members of the RPD3-superfamily contain several cysteine residues and in many cases, S-nitrosation of protein cysteine residues is conserved across the kingdoms. Human HDA2 is S-nitrosated at Cys262 and Cys274, which are located close to the catalytic center (Nott et al., 2008, 2013). A comparison of the amino acid sequence of human HDA2 and HDAs from different plant species revealed that HDA6 and HDA19 are closely related to human HDA2. The HDA domains of HDA6, HDA19 and HDA2 are highly similar (ca. 65–70% identity) whereas the C-terminal parts of the sequences are divergent (Figure 2). The highly conserved part contains the HDA domain including six highly conserved cysteine residues (Cys112, Cys163, Cys273, Cys285, Cys296, and Cys323 of *Arabidopsis* HDA6). Additionally, Cys325 of *Arabidopsis* HDA6 is highly conserved within the plant HDa6 and HDA19. The cysteine residues, which are targeted by NO in human HDA2 (Cys262 and Cys274) are located within the region, which is conserved in plant HDA6 and HDA19 (Figure 2). Moreover, structural modeling of the HDA domain of *Glycine max* HDA6 and HDA19 (HDA6 68.12% sequence identity to HDA2, *Glycine max* HDA19 69.81% sequence identity to human HDA2) based on the available crystal structure of HDA2 revealed a strikingly similar 3D-fold of these proteins, where these two conserved cysteines are located close to the substrate binding site at the same positions (Figure 3). This makes plant HDA6 and HDA19 promising candidates for NO-affected/regulated nuclear HDA isoform(s). In sum, NO-dependent regulation of plant HDAs can be considered as a key mechanism in regulation of histone acetylation and gene expression.

### Nitric Oxide Induces Expression of Demethylases and Methyltransferases and/or Affects Their Activities

Reports about the effect of NO on protein or DNA methylation in plants are rare. However, transcriptional changes in response to NO have been analyzed intensively using different techniques, for instance, cDNA-amplified fragment length polymorphism (Polverari et al., 2003), microarray (Huang et al., 2002), real-time PCR (Parani et al., 2004), and RNA-seq (Begara-Morales et al., 2014). The results of these different types of transcriptome studies demonstrated that NO is inducing a large set of genes involved in plant signal transduction, transport, defense and cell death, primary and secondary metabolism, and reactive oxygen species production and degradation. NO might regulate these genes by interacting directly with transcription factors or by regulating components of signal transduction cascades (Serpa et al., 2007; Lindermayr et al., 2010; Tavares et al., 2014; Imran et al., 2018). However, the accessibility of DNA is also an important regulatory mechanism in context of gene transcription. The accessibility of DNA can be regulated either by modification of histone tails (mainly acetylation and methylation) or by methylation of DNA. Interestingly, NO alters the expression of several methyltransferases and demethylases suggesting a regulatory role of NO in DNA and/or histone methylation (Ahlfors et al., 2009; Gibbs et al., 2014; Shi et al., 2014; Hussain et al., 2016; Kovacs et al., 2016a; Imran et al., 2018). For instance, CysNO and SNP treatment of leaves induced H3K27me3 Jumonji domain-containing histone demethylase 13 (JMJ13) expression (Ahlfors et al., 2009; Hussain et al., 2016) acting as a temperature- and photoperiod- dependent flowering repressor (Zheng et al., 2019). Enhanced endogenous levels of NO or GSNO, due to overexpression of rat neuronal NO synthase (nNOS) or knockout of GSNOR, respectively, results in downregulation of JMJ30 (Shi et al., 2014; Kovacs et al., 2016a), which demethylates H3K36me2/3, regulates period length in the circadian clock (Lu et al., 2011), and is involved in the control of flowering time (Yan et al., 2014). Transcriptomic analysis of NO-deficient *noa1-2*, *nia1nia2*, and *nia1nia2noa1-2* mutants also revealed that enzymes involved in epigenetic methylation processes are differentially expressed. For instance, chromomethylase 2 (CMT2), responsible for CHH methylation at pericentromeric heterochromatin (Stroud et al., 2013), is downregulated in each mutant (Gibbs et al., 2014). Additionally, DNA METHYLTRANSFERASE 1 (MET1) maintaining CG methylation is upregulated in *noa1-2*, but downregulated in *nia1nia2*. Further, enzymes involved in the active DNA demethylation system such as REPRESSOR of SILENCING1 (ROS1) and DEMETER-like protein 2 (DML2) (Furner and Matzke, 2011) are differently expressed in these NO-deficient mutants. Interestingly, several protein arginine methyltransferases (PRMTs) are upregulated in NO-deficient plants, for example, PRMT1a, PRMT1b, PRMT3, PRMT10, PRMT5 in *noa1-2*; PRMT1b, PRMT3, PRMT4B in *nia1nia2*; and PRMT1b, PRMT3, PRMT4B, PRMT10, PRMT5 in *nia1nia2noa1-2* (Gibbs et al., 2014). PRMT1b, upregulated in all three NO-deficient mutants, methylates H4R3 and non-histone proteins such as the RNA methyltransferase fibrillarin 2 (Yan et al., 2007). Another example is PRMT5, which catalyze symmetric dimethylation of H4R3 *in vitro* and is essential for proper pre-mRNA splicing (Deng et al., 2010). PRMT5 is upregulated in *noa1-2* and *nia1nia2noa1-2* (Gibbs et al., 2014) and positively regulated by S-nitrosylation during stress responses (Hu et al., 2017). Regarding the late flowering phenotype of these NO-deficient mutants, it is worth mentioning that the histone demethylases JMJ18 is downregulated in each mutant. JMJ18 is a H3K4 demethylase controlling flowering time (Yang et al., 2012).

NO-dependent changes in DNA-methylation have been described in rice plants exposed to 0.5 mM NO donor sodium nitroprusside (Ou et al., 2015). The treatment resulted in stress symptoms and complete growth inhibition accompanied by hypomethylation of genomic DNA predominantly at CHG sites. As a consequence, transcription of a number of genes and transposable elements was activated and expression of several genes involved in chromatin remodeling and DNA methylation homeostasis, for example, chromomethylase 3, deficient in DNA methylation 1a and 1b, and DEMETER, was disturbed (Ou et al., 2015). In these cases, DNA methylation might be regulated *via* differential expression of DNA-methyl modifiers. However, modulation of their activity by NO-based post-translational modifications cannot be excluded.

Recently, it was shown that NO regulates protein methylation during stress responses in *Arabidopsis* plants (Hu et al., 2017). The authors demonstrated that S-nitrosation of protein arginine methyltransferase 5 (PRMT5), an enzyme catalyzing symmetric demethylation of protein arginine residues, activates its enzyme activity leading to proper splicing-specific pre-mRNA of stress-related genes (Hu et al., 2017). Although this mechanism does not evolve alteration of the chromatin structure, other studies showed that PRMT5 is a highly conserved type II protein Arg methyltransferase, which amongst others interacts with and methylates histones (Hu et al., 2017). In the *prmt5-1* mutant methylation of several proteins in the range of a molecular mass of 14 kD, including histone H4 and several core components of small nuclear ribonucleoprotein of the spliceosome, was barely detectable, while this phenotype could be rescued by a PRMT5 transgene (Hu et al., 2017). Therefore, it cannot be excluded that S-nitrosation of PRMT5 also affects chromatin structure by methylating defined histone Arg residues.

In mammals, NO exposure results in decease in global 5-methylcytosine and activation of transcriptional response. This might be linked to decreased expression of DNA methyltransferases Dnmt1 and Dnmt3a. Moreover, significant changes in the methylation level of H3K9, H3K27, H3K36, and H4K20 were observed in presence of NO. These changes are well-known as important modulators of gene transcription (Vasudevan et al., 2015) and are mainly a consequence of inhibition of KDM3A/JmjC histone demethylase activities and/or increased expression of a set of histone de-methylases (KDM1, KDM3A, KDM3B, KDM4A, KDM4B, KDM4C, KDM4D, and KDM7A). JmjC histone demethylases catalyze demethylation of mono-, di-, and trimethylated lysine residues by an oxidative, Fe(II)-dependent mechanism. NO directly inhibits the KDM3A demethylase activity by forming a nitrosyl-iron complex in the active site (Hickok et al., 2013) and exposure of mammalian cells to NO resulted in a significant increase in H3K9me2, the preferred substrate for KDM3A. Furthermore, exposure of embryonic stem cells to DETA-NO caused increased H3K4me1/2/3 and H3K9me3 methylation (Mora-Castilla et al., 2010) and concentration- and time-dependent accumulation of H3K9me2 upon DETA-NO treatment of human breast carcinoma cells was reported (Hickok et al., 2013).

Eukaryotic JmjC genes are separated in 14 subfamilies: Lysine-Specific Demethylase (KDM) 3, KDM5, JMJD6, and Putative-Lysine-Specific Demethylase (PKDM) 11, and PKDM13 subfamilies present in plants, animals, and fungi. Other subfamilies are detected only in plants and animals (PKDM12) or in animals and fungi but not in plants (KDM2 and KDM4). PKDM7-9 are plant-specific groups. The existence of Jumonji C-containing histone demethylases in plants suggested that at least some might be regulated by NO, similar the ones described in mammals.

### Nitric Oxide Regulates Histone and DNA-Methylation on Metabolite Level

Methylation of histones and DNA can be regulated on two main levels–on the level of methyltransferases and de-methylases, which are catalyzing the methylation/de-methylation reactions, and on metabolite level. S-Adenosylmethionine (SAM) is the major methyl group donor in the cell. DNA and histones are subject to methylation by specific SAM-dependent methyltransferases. Each transfer of a methyl-group generates S-adenosylhomocysteine (SAH), which is cleaved into homocysteine and adenosine (Ado) by S-adenosylhomocysteine hydrolase (SAHH) (Kovacs et al., 2016b). It is known already for a long time that the equilibrium of this reversible reaction favoring SAH synthesis (Palmer and Abeles, 1976) is driven toward hydrolysis of SAH due to removal of its products (Poulton and Butt, 1976). Methionine synthase converts homocysteine to methionine, which is in turn adenylated to SAM by SAM synthetase, whereas Ado is metabolized in the Ado salvage cycle. The ratio of SAM and SAH is considered as important regulator of cellular methylation processes.

For example, Zhou et al. (2013) reported the importance of a balanced SAM/SAH ratio for DNA and H3K9me2 methylation. The defect of folate polyglutamylation caused by mutation of folylpolyglutamate synthase affects the folate-mediated one-carbon metabolism resulting in increased SAH level, reduced SAM/SAH ratio and continuatively in a reduced DNA methylation and H3K9me2. A connection between the folate cycle, DNA methylation and redox homeostasis is also reported by Groth et al. (2016). Mutation in methylenetetrahydrofolate dehydrogenase/methenyltetrahydrofolate cyclohydrolase (MTHFD1) results in loss of DNA methylation. The authors assume that reduced MTHFD1 function disturbs the cellular redox state, which might affect enzyme activities of the methylation cycle and/or methylation/demethylation reactions.

Other studies highlighted the importance of SAHH activity toward chromatin modifications (reviewed in Pikaard and Mittelsten Scheid (2014) and Vriet et al. (2015)): Mutation of the *AtSAHH1* gene resulted in reduced cytosine methylation and release of transcriptional gene silencing (Rocha et al., 2005; Mull et al., 2006; Jordan et al., 2007). Moreover, silencing of SAHH expression in tobacco plants lead to loss of DNA methylation in repetitive elements (Tanaka et al., 1997). Furthermore, in *Arabidopsis* levels of DNA and histone methylation at endogenous repeats are reduced after treatment with the SAHH inhibitor dihydroxypropyladenine (Baubec et al., 2010).

There are several hints that the supply of SAM and the removal of SAH—the by-product inhibitor of transmethyl reactions—are at least partly regulated by NO, since the activities of key enzymes of the methylation cycle seems to be modulated by NO-dependent posttranslational modifications. In several independent proteomic studies, cobalamin-independent methionine synthase, SAMS, and SAHH were identified as targets for S-nitrosation (Lindermayr et al., 2005; Abat and Deswal, 2009; Puyaubert et al., 2014; Hu et al., 2015). In *Arabidopsis,* different SAMS isoforms are differentially inhibited by protein S-nitrosation (Lindermayr et al., 2006). While isoform SAMS1 is reversibly inhibited by GSNO, the activity of the isoforms SAMS2 or SAMS3 is not affected. Responsible for the inhibition of SAMS1 is S-nitrosation of Cys114, which is located nearby the catalytic center as part of the active site loop (Lindermayr et al., 2006). In mammals, a similar regulatory mechanism of SAMS activity is described. Here two SAMS isoforms are present. While the activity of SAMS1A is reversibly inhibited by NO, SAMS2A activity is not affected (Pérez-Mato et al., 1999).

In *Arabidopsis,* two genes encode for SAHH, but only SAHH1 seems to play a role in DNA-methylation processes (Rocha et al., 2005; Vriet et al., 2015). S-Nitrosation of *Arabidopsis* SAHH1 upon cold stress was reported, but the physiological function of S-nitrosated SAHH1 is not yet investigated (Puyaubert et al., 2014; Puyaubert and Baudouin, 2014). Beside S-nitrosation, tyrosine nitration has been observed in sunflower (*Helianthus annuus L.*) SAHH. This modification decreased the catalytic activity of SAHH (Chaki et al., 2009). The results of all these different studies suggest that NO plays an important regulatory role in allocation of the major methyl-group donor SAM and the removal of the methyltransferase inhibitor SAH, which consequently affects histone and DNA methylation.

**Figure 4:**
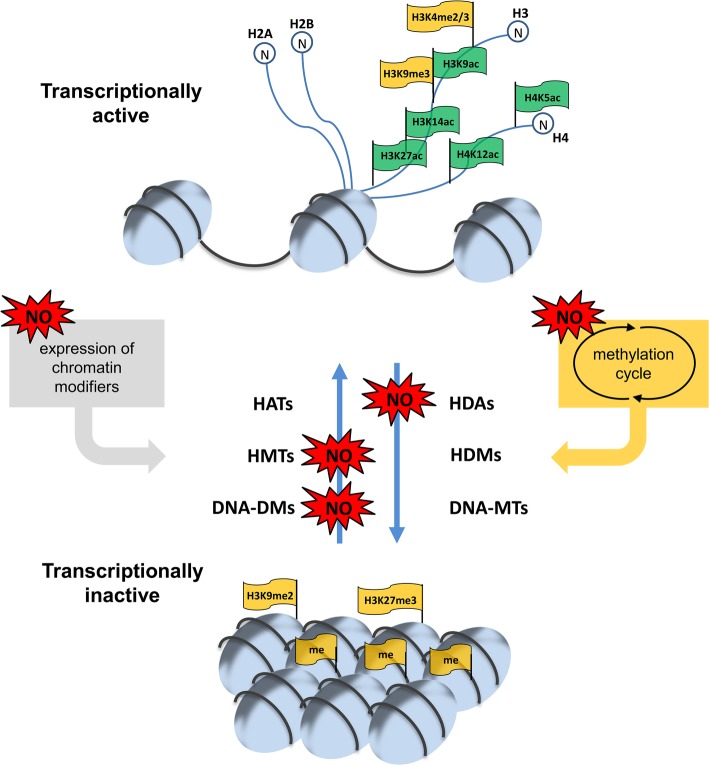
NO-dependent regulation of chromatin modulation. Histone acetylation/methylation and DNA-methylation is controlled by different sets of acetylases/deacetylases (HATs/HDAs) and methyl transferases/demethylases (HMT/HDMs and DNA-MTs/DNA-DMs). NO can regulate the expression of some of these chromatin modifiers as well as their activity. Moreover, NO can affect the supply of the methyl group donor SAM and the level of the methyltransferase inhibitor SAH by altering the activity of enzymes of the methylation cycle and/or connected pathways. For more details see this paper.

In sum, there are different levels, where NO can affect chromatin modulation, for instance transcription and activity of chromatin modifiers or the supply of methyl group donors or methylation inhibitors (summarized in Figure 4). Since NO is an important signaling molecule in plant growth and development and in plant stress response all these different mechanisms discussed above allows NO to regulate physiological processes. The most important future challenges are the identification and/or verification of NO-regulated chromatin modifiers responsible for histone modifications and DNA methylation and the characterization of the mode of action of NO on these proteins. Moreover, the corresponding histone marks and the chromatin regions controlled by NO-regulated chromatin modifiers have to be identified. Such analysis would also include possible interaction between histone marks and DNA-methylation. Additionally, these NO-dependent chromatin modifications have to be analyzed in a physiological context to complement the picture of NO signaling function in plants.

Finally, the gained knowledge could be subjected to genetic/epigenetic engineering or classical breeding to improve plant traits permanently.

## Author Contributions

AA-K, ER and CL wrote the review article. CL coordinated the completion of the article.

### Conflict of Interest Statement

The authors declare that the research was conducted in the absence of any commercial or financial relationships that could be construed as a potential conflict of interest.
